# Assessment and Modeling of Plasmonic Photothermal Therapy Delivered via a Fiberoptic Microneedle Device Ex Vivo

**DOI:** 10.3390/pharmaceutics13122133

**Published:** 2021-12-10

**Authors:** Forhad Akhter, Santiago Manrique-Bedoya, Chris Moreau, Andrea Lynn Smith, Yusheng Feng, Kathryn M. Mayer, R. Lyle Hood

**Affiliations:** 1 Department of Mechanical Engineering, University of Texas at San Antonio, San Antonio, TX 78249, USA; forhad.akhter@utsa.edu (F.A.); santiago.manriquebedoya@utsa.edu (S.M.-B.); andrea.smith@my.utsa.edu (A.L.S.); Yusheng.Feng@utsa.edu (Y.F.); 2 Gastroenterology and Transplant, UT Health San Antonio, San Antonio, TX 78229, USA; MoreauC@uthscsa.edu; 3 Department of Physics & Astronomy, University of Texas at San Antonio, San Antonio, TX 78249, USA; Kathryn.Mayer@utsa.edu; 4 Graduate School of Biomedical Sciences, UT Health San Antonio, San Antonio, TX 78229, USA

**Keywords:** plasmonic photothermal therapy, gold nanorods, fiberoptic microneedle, near infrared, photothermal, COMSOL tissue model, bioheat, hyperthermia

## Abstract

Plasmonic photothermal therapy (PPTT) has potential as a superior treatment method for pancreatic cancer, a disease with high mortality partially attributable to the currently non-selective treatment options. PPTT utilizes gold nanoparticles infused into a targeted tissue volume and exposed to a specific light wavelength to induce selective hyperthermia. The current study focuses on developing this approach within an ex vivo porcine pancreas model via an innovative fiberoptic microneedle device (FMD) for co-delivering light and gold nanoparticles. The effects of laser wavelengths (808 vs. 1064 nm), irradiances (20–50 mW·mm^−2^), and gold nanorod (GNR) concentrations (0.1–3 nM) on tissue temperature profiles were evaluated to assess and control hyperthermic generation. The GNRs had a peak absorbance at ~800 nm. Results showed that, at 808 nm, photon absorption and subsequent heat generation within tissue without GNRs was 65% less than 1064 nm. The combination of GNRs and 808 nm resulted in a 200% higher temperature rise than the 1064 nm under similar conditions. A computational model was developed to predict the temperature shift and was validated against experimental results with a deviation of <5%. These results show promise for both a predictive model and spatially selective, tunable treatment modality for pancreatic cancer.

## 1. Introduction

Pancreatic ductal adenocarcinoma (PDAC) is one of the most aggressive and lethal malignancies with a median patient survival of 6–8 months after diagnosis and a five-year mean survival rate of around 10% [1,2]. It is predicted to be the second largest cause of cancer related death in the US by 2030 with a current mortality rate of 42,000 death per year [2]. Despite substantial advancements in medical science, efforts in early diagnosis techniques, and innovative therapeutic approaches, survival rates have not improved significantly [2]. A majority of the patients (60–70%) are not surgical candidates due to advanced age, late-stage diagnosis, or progression of cancer with proximity to major vasculature [3,4]. Non-surgical techniques including chemo- and radiotherapy have been proven to have low effectiveness in pancreatic cancer due to poor specificity and adverse side effects [5,6,7]. Minimally invasive and selective therapeutic approaches are desperately needed to help improve patient outcomes.

Endoscopic ultrasound (EUS) has become the gold-standard for diagnosing pancreatic cancer due to its ability to provide high resolution imaging and a means to access the lesion for biopsy or therapeutics [7]. EUS-guided radiofrequency ablation (RFA) techniques utilizing a catheter directly inserted into the tumor have been proven to be feasible, although with significant rates of pancreatitis [8]. EUS-guided laser ablation (laser interstitial thermal therapy; LITT) in a healthy pancreas has recently been successfully demonstrated in a pig model and a pilot clinical trial, but controlling the ablation zone was an issue due to tissue non-selectivity [6,9]. Without tissue-selective and tumor-directed therapies, the risk of normal tissue damage and subsequent complications such as pancreatitis remains high.

A promising emerging technology for the treatment of unresectable pancreatic cancer is plasmonic photothermal therapy (PPTT) [10,11]. The process involves infusing gold nanoparticles (GNPs) to the targeted tumor region followed by exposure to near-infrared light (NIR, 750–1700 nm) to induce hyperthermia and tissue damage [12,13,14]. The free electrons of GNPs resonate and convert energy to heat when exposed to a specific light wavelength based on the geometry of the GNPs in a phenomenon known as surface plasmon resonance [11,15]. The effect seems highly useful for cancer therapy, as it can be tuned through modification of the size and shape of the GNPs during synthesis [11,16]. Costantini et al. reported the application of branched gold nanoparticles (BGNPs) in colon cancer cells for the photothermal therapy at 808 nm laser [17]. In addition, 2D nanomaterials including Pd-Au nanoplates showed promise in photothermal therapy due to their high light to heat conversion rate, optical tunability through integration of other plasmonic metals, and large surface area accommodating other molecules for synergized therapy [18]. Despite numerous previous studies exploring PPTT for other diseases, the application of this technique for the treatment of pancreatic cancer is largely unexplored [19,20,21,22]. What little work exists has explored ex vivo benchtop and early in vivo models, but demonstrated some promise that GNP photothermal heating can destroy malignant cells and inhibit tumor growth [19,23]. Gold nanorods (GNRs), among other GNP geometries, are increasingly utilized in PPTT due to their potential for high plasmonic heat generation [16,24] and optical dose tunability [25,26,27]. GNRs have been applied in numerous ex vivo and in vivo models of other varieties of cancer to explore the efficacy of PPTT [11,13]. Pulagam et al. reported an in vivo application of radiochemically stable GNRs in a mouse xenograft model for the photothermal therapy of gastrointestinal cancer [28]. GNRs can be functionalized to localize specifically within and around a tumor volume [21,29]. A recent study by Polo et al. demonstrated that plasmonic heating significantly changes the composition of protein corona around the GNRs which can be manipulated through surface charging of PEGylated GNRs [30]. However, the clinical application of PPTT for pancreatic cancer treatment will require precise control of GNR localization and thermal ablation, requiring further research to realize.

A fiberoptic microneedle device (FMD) was developed by our group through leveraging an innovative light fluid coupling technique for co-delivering high power laser light (both visible and NIR) and liquid solutions (e.g., GNR solution) to a targeted tissue volume [30,31,32,33]. The sharp FMD tip is capable of penetrating soft tissue to infuse fluids and deliver light either peri- or intra-tumorally, reducing the chance of damaging healthy tissue in the optical path. Prior work also demonstrated the local photothermal heating with 1064 nm wavelength resulted in deep penetration and controlled volumetric dispersal of macromolecules in rat cerebral and porcine bladder tissue [34,35]. These phenomena inspired deploying FMD, guided through EUS, for the application of PPTT in pancreatic cancer treatment.

A computational model for predicting the tissue–GNRs interaction with different light wavelengths would greatly assist in determining a safe and effective PPTT approach for pancreatic cancer. A literature review revealed numerous finite element (FEM) analyses describing tissue photothermal heating by NIR light [36,37,38,39]. However, only a few reported the FEM model describing the porcine pancreas tissue–NIR light (1064 nm only) interaction [24,39]. However, these models utilized porcine liver tissue thermal and optical properties instead of pancreatic tissue due to a gap in the literature on this topic [39]. Moreover, porcine pancreas interaction with ~800 nm range wavelength has not been reported yet. A computational model was developed earlier by our group to characterize light absorption and photothermal heating of GNRs by the NIR light in atmospheric condition, setting the stage for a surgical planning package [24]. However, the laser–pancreas tissue interaction was not reported in that study due to the gap in the literature. Another recent work of our group focused on bridging this gap in the literature by reporting porcine pancreas thermal and optical properties [40]. The current study utilized these tissue specific properties and reported a computational model for the photothermal heating of porcine pancreas tissue through collimated laser and FMD at 808 and 1064 nm. As the porcine pancreas tissue is analogous to humans and a suitable candidate for ex vivo modeling [41,42], a computational model on NIR light interaction with porcine pancreas tissue would benefit both researchers and clinicians exploring treatments for pancreatic cancer.

The aim of this study is to characterize photothermal heating of ex vivo porcine pancreas tissue at two different wavelengths, 808 and 1064 nm. Light delivery is explored with both a free beam and through the FMD, both with and without the presence of GNRS. A computational model was developed utilizing tissue-specific thermal and optical properties to predict the tissue temperature profile when exposed to 808 and 1064 nm light. A comparison study between experimental and computational results was reported to validate the model.

## 2. Materials and Methods

### 2.1. Tissue Sample Collection

Porcine pancreas tissue samples were obtained from a USDA-approved abattoir. Immediately after the animals were sacrificed (<10 min), the pancreas was excised, and placed in separate Ziploc bags. An insulated cooler box with ice was used for transporting the samples to the laboratory. Upon arrival at the laboratory, the tissue samples were washed in phosphate-buffered saline (PBS) solution. Samples were sliced in a 3 × 3 cm^2^ cross-section area using a scalpel and the dimensions were estimated using calipers and image processing software (ImageJ) [43]. Samples used in different experiments had a thickness of approximately 1 ± 0.05 cm. A portion of these tissue specimens was utilized in running experiments on the same day of collection (referred to as ‘fresh’ tissue samples). The rest of the specimens were stored individually in a −62 °C freezer for later use within 7–14 days (referred to as ‘frozen’ tissue samples). All study procedures were completed following the approved protocols by the University of Texas at San Antonio’s Institutional Biosafety Committee.

### 2.2. GNRs Synthesis and Photothermal Heating

GNRs were synthesized by a seed-mediated growth protocol described in the literature [25,44,45]. The detailed method of the synthesis process was described previously by this group [24]. The optical absorbance of the nanorods was confirmed via UV-Vis spectroscopy (400–1100 nm) and their geometry (length, width, aspect ratio) was measured via SEM (scanning electron microscopy) imaging and analyzed in ImageJ. As-synthesized GNRs (i.e., non-functionalized) were utilized in this experiment where GNRs were suspended in CTAB solution (Cetyltrimethylammonium bromide). The initial concentration of the solution was 3.2 nM, which was evaluated from the mass percentage of gold in a known volume of GNR solution (3.5 mL cuvette). The mass percentage was measured by quantifying the weight of gold through centrifuging the solution and subtracting the weight of water. This concentrated solution was serially diluted by adding 10 mL of distilled water in each increment. The optical absorbance of each diluted solution was obtained from the UV-Vis spectrometer. An absorbance vs. GNR concentration graph was plotted from these optical measurements which was the basis for identifying any unknown GNR concentration from the optical absorbance measurement (see Figure S1 in Supplementary Materials).

**Figure 1:**
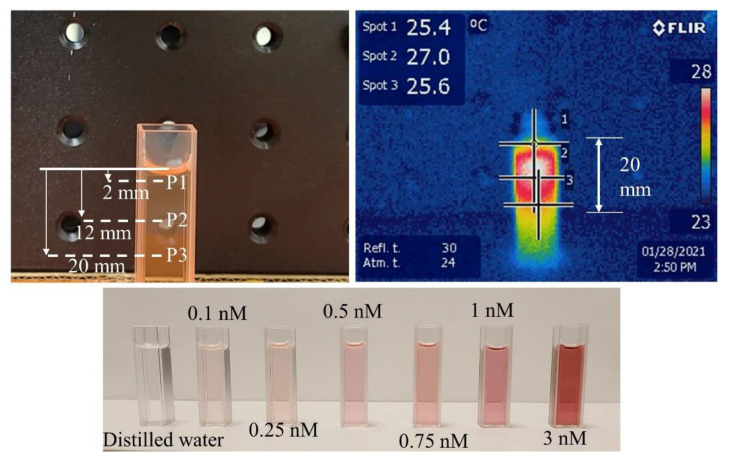
Gold nanorods (GNRs) photothermal heating with 808 nm laser.

Next, the maximum steady state temperatures of different GNR concentrations (0.1, 0.25, 0.5, 0.75, 1, 3 nM) were measured at fixed laser irradiations (30 mW·mm^−2^ at 808 and nm). The experimental setup included a transparent cuvette (3.5 mL) filled with a known concentration of GNR solution exposed under the collimated laser beam (Figure 1). The distance between the laser pointer lens and the GNR top surface was fixed at 3 cm. The cuvette cross-sectional area (100 mm^2^) was significantly larger than the collimated beam area (24 mm^2^), which ensured the unobstructed interaction between the laser and GNRs. A thermal camera (E5, FLIR, Wilsonville, OR, USA) was positioned 30 cm away to capture a lateral image of the cuvette and laser. The camera setting was adjusted to measure temperature at three different spots on the cuvette: 2 mm, 12 mm, and 20 mm below the top surface of the GNR solution (as shown in Figure 1). The purpose was to observe the temperature distribution as a function of depth into the GNR solution and evaluate the mean value of these three measurements. The thermal camera was connected to a computer for real-time data collection at 5 Hz. Laser irradiation continued until the GNR temperature reached a steady state (temperature change <0.05 °C for 5 min). The experiment was repeated 5 times for each concentration of the solution (*n* = 5). A control test was conducted following a similar procedure with distilled water (blank) for comparison.

### 2.3. Tissue Photothermal Heating with Collimated Laser Beam

**Figure 2:**
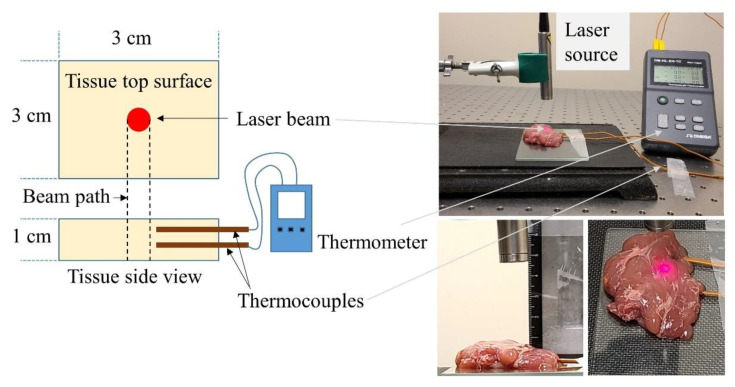
Experimental setup for the ex vivo porcine pancreas tissue photothermal heating by 808 and 1064 nm collimated laser beams. Note that the red guide beam is visible but is not the experimental light source.

The photothermal heating experiments utilized two different continuous wave laser sources with collimated beam outputs at 808 nm (LRD-0808-PFR-01000-03, Laserglow Technologies, Toronto, ON, Canada) and 1064 nm (YLR 10-1064-LP, IPG photonics, Oxford, MA, USA) wavelengths. The collimated beam areas for both laser sources were estimated by taking a thermal image (E40, FLIR thermal camera) of the beam reflection and post-processing the image using ImageJ. The beam areas were measured at 24 and 19.6 mm^2^ for the 808 and 1064 nm, respectively. Collimated beam output power was measured by an integrating sphere (photo detector, 819D-UV-2-CAL, Newport, Franklin, MA, USA) and an optical power meter (1936-R, Newport, Franklin, MA, USA). The beam areas were used to set the beam intensity within the range of 20–50 mW·mm^−2^ (in 10 mW·mm^−2^ increments) for both laser sources. This range was selected through a set of preliminary tests with different laser irradiations which demonstrated that >60 mW·mm^−2^ irradiation would cause unwanted tissue burning (see Figure S2 in Supplementary Materials). The tissue specimen was placed on a glass slide (12 × 12 × 0.5 cm) beneath the laser attached to an adjustable holder (Figure 2). The distance between the tissue top surface and the laser was kept constant at 4 cm. Two K-type thermocouples (Fluke Co; Everett, WA, USA) were inserted into the tissue at 3 and 6 mm below the tissue surface while ensuring they were 1–2 mm from the collimated beam path. An Omega thermometer (OM-HL-EH-TC, Omega Engineering, Norwalk, CT, USA) was utilized to record the temperature reading from the thermocouples at a set frequency of 5 Hz. Both thermocouples were calibrated against a cold junction (ice) before conducting the experiments. A glass thermometer was used to measure the room temperature. The experiment started when the tissue’s initial temperature reached room temperature (22 °C). Laser emission continued until the tissue temperature reached a steady state maximum range, i.e., temperature fluctuation was <0.05 °C for 5 min. At this point, the laser was turned off to allow the tissue specimen to cool normally (no forced convection) to room temperature. The experiment was repeated 5 times with different specimens (*n* = 5). A similar procedure was followed for experiments with two different laser sources and four different light intensities.

### 2.4. Tissue Photothermal Heating with FMD and with/without the GNRs

**Figure 3:**
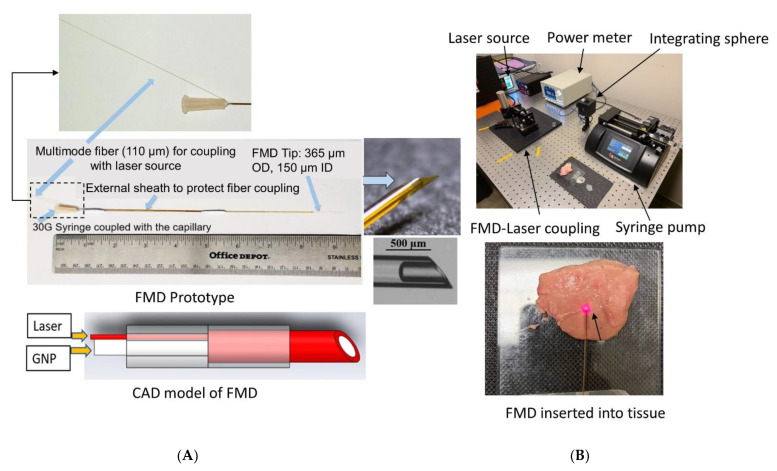
(**A**) Fiberoptic microneedle device (FMD) (Reproduced with permission from [32], Elsevier, 2020 and (**B**) the experimental setup for tissue photothermal heating with FMD at 808 and 1064 nm wavelengths.

The fabrication process, optical performance, and mechanical characterization of the FMD were described previously by members of this group [31]. In brief, the FMD utilized a flexible light guiding capillary (fused silica, 365 μm outer diameter, and 150 μm inner diameter) which can co-deliver light (both visible and NIR) through the annular silica core and liquid through the hollow bore (Figure 3). The tip of the FMD can be either flat polished or beveled at a specific angle for easy penetration of soft tissue. The device was designed for connecting to a laser source and a syringe for co-delivering light and fluid, respectively. The goal of using FMD in this study was to assess its utility as a delivery vehicle for GNRs and thereby PPTT. The cross-section area of the annular silica core of a flat polished FMD tip was evaluated from capillary geometry (0.086 mm^2^) to identify the applied laser intensity. FMD tips were inserted horizontally (2–3 mm) into the tissue top surface lying on a glass slide at room temperature (22 °C) (Figure 3). The laser exposure time of 60 s was decided upon through discussion with clinical collaborators at the University of Texas Health Science Center at San Antonio. The aim was to complete the procedure in a short time to avoid the unwanted heating of healthy surrounding tissue. A set of preliminary tests were conducted to evaluate the threshold light intensity from the FMD tip that would induce tissue carbonization, which would be an unwanted outcome to avoid. It was observed from the preliminary tests that laser intensities >60 mW·mm^−2^ from the FMD tip for both 808 and 1064 nm wavelengths could cause tissue carbonization within the 60 s exposure period. Hence, the FMD tip output laser intensity was set sequentially at 30, 40, and 50 mW·mm^−2^ for this test. A thermal camera was positioned above the tissue sample at a fixed height of 30 cm to capture the tissue temperature profile (Figure S4 of Supplementary Materials shows the thermal camera images taken during the experiments). The camera was connected to a computer for recording temperature data at a 2 Hz sampling rate. After 60 s, the laser was turned off, and the tissue was allowed to cool down to room temperature. The test was repeated five times with different tissue samples for each light intensity at both 808 and 1064 nm (*n* = 5).

#### GNR Infusion and Tissue Photothermal Heating through FMD

Another set of experiments were conducted to assess the combined effect of laser irradiation and GNR concentration on ex vivo porcine pancreas tissue photothermal heating. The experimental setup was similar to the previous experiments except for the addition of the GNR solution transfused through the FMD by a syringe pump (Figure 3). The FMD was also coupled by a free coupler to the 808 or 1064 nm laser, which was delivered at the same irradiances as before (30, 40, and 50 mW·mm^−2^). GNR concentrations infused included 0.1, 0.25, 0.5, 0.75, and 1 nM delivered at 1 mL/min for 60 s. Laser irradiation initiated right after the infusion of GNRs at 60 s. Tissue temperature was monitored through the thermal camera following the same instruction as mentioned earlier. Laser irradiation continued for 60 s followed by the natural convective cooling to room temperature (22 °C). The experiment was repeated five times for each laser irradiation and GNR concentration (*n* = 5). The tissue specimen was replaced between each experiment.

### 2.5. Computational Modelling of Laser–Tissue Interaction 

**Figure 4:**
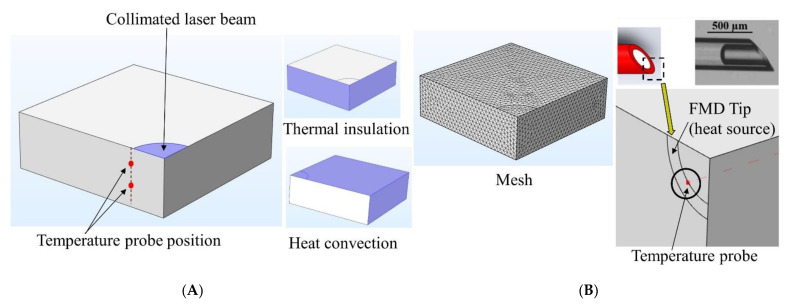
Computational modelling of ex vivo tissue photothermal heating by (**A**) collimated beam and (**B**) FMD. From left to right: probe positioning for the collimated beam model, boundary conditions imposed on the model, computational mesh used for FEA, FMD model, and probe position.

A computational model was developed to predict the tissue temperature rise induced by photothermal heating at the 808 nm and 1064 nm wavelengths. The modelling procedure was adapted from Feng et al. [38,46] and Saccomandi et al. [39] with the inclusion of the tissue-specific thermal and optical properties at both laser wavelengths from our previous work [40]. The 3D model was developed using the FEA software COMSOL Multiphysics^®^ with a 3 × 3 × 1 cm^3^ block representing the tissue sample and collimated laser beam simulated by a circular area at the center of the block. Due to axial symmetry, only a quarter of the model is required to perform the simulations (Figure 4).

The effect of photothermal heating induced by the laser beam can be modeled by the Pennes bioheat transfer equation [47]. Assuming the model is a representation of laser heating in ex vivo tissue, the blood perfusion and metabolism contributions can be neglected. Thus, the bioheat equation reads:

(1) $${\rho C}_{p}\frac{\partial T\left(x,t\right)}{\partial t}-\nabla .\left(k\nabla T(x,t\right)=Q_{\mathrm{light}}\left(x,T\right)$$

Here, ρ (kg⋅m^−3^), $C_{p}$ (Jkg^−1^K^−1^), and k (Wm^−1^·K^−1^) are the tissue density, specific heat, and thermal conductivity, respectively. For simplicity, the tissue was considered homogeneous and isotropic. T(x,t) is the tissue temperature, expressed as a function of space and time. $Q_{\mathrm{light}}\left(x,T\right)$ (W·m^−3^) is the heat source term due to photon absorption caused by the laser–tissue interaction, which can be expressed as follows [38]:

(2) $$Q_{\mathrm{light}}\left(x,T\right)={µ}_{a}.\Phi \left(x,t\right).E$$

Here, ${µ}_{a}$ (mm^−1^) is the absorption coefficient of tissue at a specific laser wavelength, Φ(x,T) (m^−2^·s^−1^) is the photon fluence (number of photons passing through a unit area at a point in space per unit time), and E (J) is the photon energy. The photon energy can be evaluated as

(3) $$E=\frac{\mathrm{hc}}{\lambda }$$

with h (6.63 × 10^−34^ J·s) being Plank’s constant, c (2.99 × 10^8^ m·s^−1^) the speed of light, and λ (m) the laser wavelength. Additionally, the photon fluence in the tissue (i.e., Φ(x,t) in Equation (2) can be estimated from the time-dependent light diffusion approximation derived from the radiative transfer equation [48]. With no additional sources apart from light absorption, the equation reads:

(4) $$\frac{1}{c}\frac{\partial }{\partial t}\Phi \left(x,t\right)+\nabla .\left(-D\nabla \Phi \left(x,t\right)\right)=-{µ}_{a}.\Phi \left(x,t\right)$$

Here, ${µ}_{a}$ (m^−1^) is the absorption coefficient of tissue at a specific light wavelength, and D is the optical diffusion coefficient which depends on tissue-specific optical properties. The diffusion coefficient can be expressed as [49,50]:

(5) $$D=\frac{1}{\left[3({µ}_{a}+{µ}_{s}^{\prime })\right]}$$

where ${µ}_{s}^{\prime }$ (m^−1^) is the reduced scattering coefficient of tissue at a specific laser wavelength. 

Equations (1) and (4) were solved using the heat transfer and general form PDE modules in COMSOL Multiphysics^®^, respectively. Tissue initial temperature (T|_t = 0_) was set at 22 °C to mimic the ex vivo experiment. The collimated laser beam on the tissue surface was modeled using a Dirichlet boundary condition [51]:

(6) $$\Phi {|}_{\mathrm{source}}=\left(1-R\right)\frac{w_{0}}{{\pi r}_{0}^{2}\left(1-E\right)}$$

with R being the light reflectance at the air–tissue interface for a specific laser wavelength, $w_{0}$ (W) the laser power, and $r_{0}$ (m) the collimated beam radius. In the current model, the laser power was assumed to be uniform over the source boundary. Thermal insulation boundary conditions (Neumann boundary condition [51]) were imposed at the symmetry planes (left and front boundaries). Lastly, free convective cooling ($q_{\mathrm{conv}}$) was imposed at the top, right, and back boundary area of the tissue (Figure 4).

(7) $$q_{\mathrm{conv}}=h_{\mathrm{conv}}\left(T\left(x,t\right)-T_{\infty }\right)$$

Here, $h_{\mathrm{conv}}$ (5 Wm^−2^·K^−1^) is the convection heat transfer coefficient, and $T_{\infty }$ (295.15 K) is the ambient temperature. Furthermore, porcine pancreas tissue properties were obtained from both literature (e.g., ρ=1040 kg·m^−3^ and $C_{p}=3630$ J·kg^−1^·K^−1^) [39,52] and our team’s previous work on characterization of tissue thermal and optical properties (e.g., k=0.45 Wm^−1^·K^−1^, ${µ}_{a}$, ${µ}_{s}^{\prime }$, and R (detailed optical properties are shown in Table S1 of Supplementary Materials)) [40]. After setting up the equations and boundary conditions, two temperature probes were placed at 3 and 6 mm below the tissue’s top surface and 2 mm away from the beam path to mimic the experimental setup described in Section 2.2 (Figure 4). The model was run to assess the steady state tissue temperature for a 0–60 min period when exposed to different laser irradiations (20, 30, 40, 50 mW·mm^−2^) at both 808 and 1064 nm wavelengths.

To assess the effect of photothermal heating using the FMD, the collimated beam area of the model was replaced by the annular core of the FMD (Figure 4). It was assumed that photons are uniformly distributed throughout the core area of the FMD and scattered at the tip. A quarter of the FMD tip was modeled using the same procedure described earlier for the collimated beam. Uniform laser power over the source boundary was assumed. The rest of the boundary conditions remained the same. The model was run for 60 s using 30, 40, and 50 mW·mm^−2^ laser irradiations at both 808 and 1064 nm. Finally, the simulation results were compared with the experiments as described earlier in Section 2.3 and Section 2.4 to validate the model.

## 3. Results and Discussion

### 3.1. Results of GNRs’ Synthesis and Photothermal Heating

**Figure 5:**
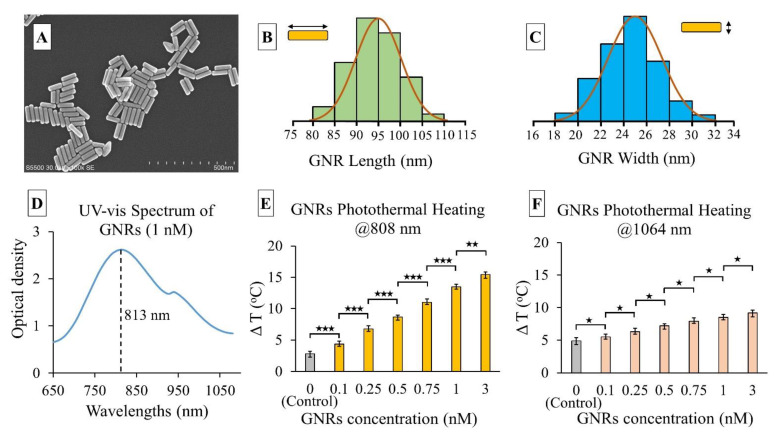
(**A**) SEM image of the GNRs; representation of GNRs’ size-frequency histogram of *n* = 100 distribution as determined from SEM image analysis: (**B**) GNR length (95.2 ± 4.7 nm), (**C**) GNR width (24.8 ± 1.5 nm), average aspect ratio (length/width): 3.83; (**D**) UV-vis absorption spectrum of a sample GNR solution (1 nM concentration) with an absorption peak at 813 nm wavelength; temperature rise (∆T) vs. GNR concentration graph when exposed to 30 mW·mm^−2^ beam at (**E**) 808 nm, and (**F**) 1064 nm laser wavelengths. The error bars represent mean ± 1σ (standard deviation) for *n* = 5 trials. Student’s T-test showed the statistical significance between two consecutive GNRs’ concentrations and control (distilled water) which are represented by ★ symbol. ★ = *p* < 0.05, ★★ = *p* < 0.01, and ★★★ = *p* < 0.001.

SEM images of the GNRs were utilized to obtain the average dimension of nanorods (Figure 5). The measured length and width of the GNRs were 95.2 ± 4.7 nm and 24.8 ± 1.5 nm respectively. A spectrometric analysis showed the resonance wavelength (peak absorbance) of the GNRs to be 813 nm. At 1064 nm, the GNRs’ optical absorbance reduced to less than half of the peak absorbance. Theoretically, these GNRs should exhibit high photon absorption and heat generation at 808 nm which was assessed in this experiment. In the photothermal heating experiment of different GNR concentrations (0.1–3 nM) at 808 and 1064 nm, the 30 mW·mm^−2^ collimated laser beam showed different temperature gradients (Figure 5). A statistical analysis (Student’s T-test) was conducted between the results from each set of two consecutive GNRs concentrations to assess significance. It was observed that at 808 nm, the significance was higher (*p* < 0.001) for concentrations ranging from 0.1 to 1 nM. The maximum temperature did not increase as dramatically from 1 to 3 nM concentrations (*p* < 0.01). This may be attributable to the saturation of photon absorbance by GNRs at a higher concentration for a fixed power density. In contrast, at 1064 nm, GNR temperature did not increase as much as the 808 nm wavelength, though the control test with distilled water demonstrated a significant increase in ∆T (4.8 °C). This could be attributed to the higher light absorption rate of water at the 1064 nm range. The thermal camera captured temperatures at different spots of the cuvette filled with GNRs. During the experiment, it was observed that maximum heat was generated at a 12 mm depth from the top surface of the GNR solution in the cuvette (Figure 1). GNRs absorbed more light and gradually increased the temperature within this top layer as the concentration increased. Most of the light scattering and absorption occurred within the upper layer (~20 mm) of GNRs while the bottom of the cuvette showed very little temperature increase (1–2 °C). A low concentration (0.1 nM) GNR solution raised the temperature (∆T) by 5 °C, which increased almost three-fold for the 1 nM solution. The rate of temperature rise was directly proportional to the GNR solution concentration. The 3 nM solution rapidly increased the temperature by 10 °C within 1 min, a rate which may be too fast to control during the application of PPTT. Choosing the correct GNR concentration and laser exposure will be critical for generating sufficient heat to cause tissue hyperthermia while minimizing damage to nearby healthy tissues. This experiment demonstrated the light-to-heat conversion efficiency of different GNRs’ concentrations at 808 nm, the targeted wavelength for PPTT application in this study.

### 3.2. Results of Tissue Photothermal Heating by Collimated Laser Beam

**Figure 6:**
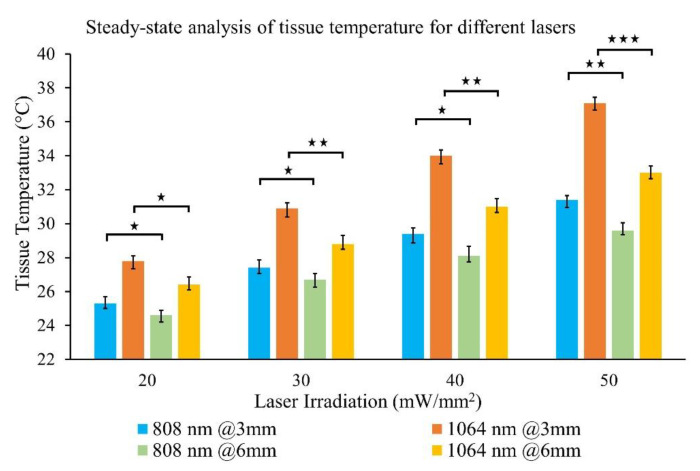
Steady-state temperature of ex vivo porcine pancreas tissue at 3 mm and 6 mm depth from top surface during photothermal heating at different laser irradiations (20, 30, 40, 50 mW·mm^−2^) of both 808 and 1064 nm wavelengths. The error bar represents mean ± 1σ (standard deviation) for *n* = 5 trials. Statistical analysis (T-test) was conducted between results from 3 mm and 6 mm tissue depths for a fixed laser wavelength and irradiation. The significance of the data is represented by ★ symbol where ★ = *p* < 0.05, ★★ = *p* < 0.01, ★★★ = *p* < 0.001.

In this experiment, ex vivo tissue samples were photothermally heated by 808 and 1064 nm laser irradiation within the 20–50 mW·mm^−2^ range with a 50% increment rate. Thermocouples inserted into the tissue detected temperatures at 3 and 6 mm depth from the top surface. Maximum steady-state temperature and time were recorded for each laser irradiance. Results from the separate sets of experiments were compiled together in a single plot to facilitate the comparison of tissue photothermal heating at a fixed depth and laser irradiation for both wavelengths (Figure 6). Figure 6 represents the mean value of five experimental repetitions with an error bar representing one standard deviation (1σ). A graph showing the tissue temperature fluctuations vs. time is exhibited in Supplementary Materials (Figure S3). 

An analysis of the results revealed that the tissue temperature increase (∆T = difference between the tissue initial and final temperature) at 808 nm was lower than 1064 nm wavelength for similar irradiations: 51.4 ± 5.5% and 65.8 ± 3.2% lower at 3 mm and 6 mm tissue depths, respectively. ∆T increased linearly with respect to laser power for both wavelengths. The maximum ∆T was 15.1 °C for 1064 nm, 50 mW·mm^−2^ irradiances at 3 mm tissue depth. No visible tissue damage was observed during the experiment. For similar laser irradiations, the rate of temperature increase (∆T/time for tissue heating) was 95.6 ± 4.8% and 87.7 ± 6.1% higher for 1064 nm relative to 808 nm at 3 and 6 mm tissue depths, respectively. This finding is in good agreement with literature reports and prior work from our group on porcine pancreas tissue optical properties measurements [15,16,42]. It is evident from Figure 6 that low irradiation by 808 nm, i.e., 20/30 mW·mm^−2^ would not increase the tissue temperature as much as 1064 nm. It also implies that if the laser exposure time is reduced, the 808 nm wavelength would be less harmful to the healthy surrounding tissue than the 1064 nm. Hence, the 808 nm will be more selective for the photothermal ablation of pancreas tissue than the 1064 nm when paired with functionalized GNRs of the appropriate resonance to specifically target the tumor region only. The 1064 nm wavelength may be helpful for approaches wherein deep penetration and rapid temperature rise are desirable.

### 3.3. Results of Tissue Photothermal Heating by FMD and with/without GNRs

**Figure 7:**
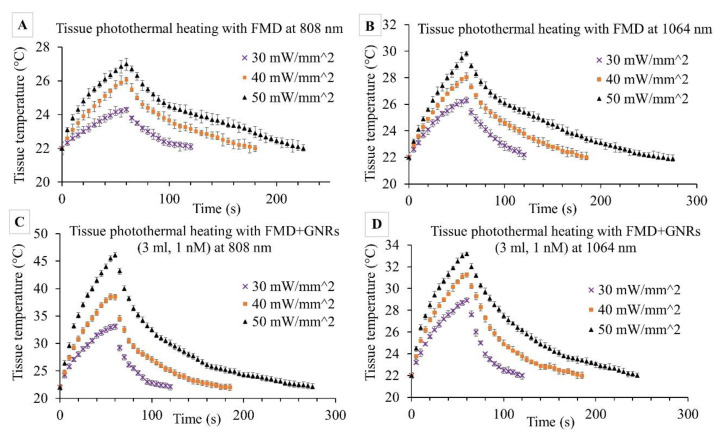
Ex vivo porcine pancreas tissue temperature profile during photothermal heating by FMD only without GNRs at (**A**) 808 nm, (**B**) 1064 nm; and with infused GNRs (1 nM, 1 mL) at (**C**) 808 nm, (**D**) 1064 nm wavelengths. Three different laser irradiations were tested: 30, 40, and 50 mW·mm^−2^ with an exposure time of 60 s followed by free convection cooling. Error bars represent mean ± 1σ (standard deviation) for *n* = 5 trials. Statistical analysis (Student’s T-test) between the maximum tissue temperatures of each set of two consecutive laser irradiations for a given wavelength showed the significance of the data (*p* < 0.01).

The purpose of this set of experiments was to evaluate the FMD’s effectiveness in the photothermal heating of ex vivo porcine pancreas tissue with and without a local GNR solution. The small size of the FMD tip (silica core area = 0.25 mm^2^) focused the high-energy laser to the specific tissue area, which resulted in rapid and concentrated heating. Concentrated irradiation caused tissue burning and carbonization at the FMD tip when the applied laser intensity was >60 mW·mm^−2^ or the exposure time was too long. Preliminary tests established the range of laser intensities (30–50 mW·mm^−2^) and exposure times (60 s) for both 808 and 1064 nm wavelengths to avoid tissue burning at the FMD tip. The results of these experiments are illustrated in Figure 7A–D. The first set of experiments (Figure 7A,B) evaluated FMD irradiation without GNRs while the next set (Figure 7C,D) explored irradiation with locally infused GNRs.

Initial irradiation experiments without GNRs exhibited faster rates of temperature increase (∆T/time) for 1064 nm wavelengths than the 808 nm at different intensities. For 808 nm, ∆T after 1 min was measured at 2.3 ± 0.6, 4.1 ± 0.4, and 5.1 ± 0.5 °C for 30, 40, and 50 mW·mm^−2^ irradiances, respectively. For similar irradiances at 1064 nm, ∆T was almost double (4.3 ± 0.4, 6.1 ± 0.7, and 8.2 ± 0.6 °C). These results followed a similar trend as observed in previous experiments with collimated beam photothermal heating (Section 3.2) for similar laser irradiations. The differences between the photothermal heating through the collimated beam and FMD are the photons distribution and the exposed tissue area. The collimated beam uniformly distributes the photons over a larger area compared to the FMD tip where photons spread out in a much smaller tissue area resulting in a rapid temperature increase. During the application of the PPTT, this phenomenon will help in selectively heating a tissue volume of interest.

The next set of experiments included the local infusion of the GNR solution prior to irradiation with the same wavelengths and irradiances as the prior set of experiments. The GNR concentrations were increased (0.1, 0.25, 0.5, 0.75, and 1 nM) while keeping the total infused volume constant (1 mL). Figure 7C,D exhibits the results for 1 nM GNR solution, as it provides a good representation of the observed trends. At 1064 nm, ∆T increased by 2.6 ± 0.4, 3.1 ± 0.5, and 3.6 ± 0.3 °C due to the inclusion of GNRs at 30, 40, and 50 mW·mm^−2^, respectively. In contrast, at 808 nm, ∆T increased significantly by 8.8 ± 0.5, 12.5 ± 0.5, and 19.1 ± 0.6 °C while comparing the results between Figure 7A,C for similar irradiances. Following 808 nm exposure to pancreatic tissue with infused GNRs, visible bleaching around the FMD insertion area was evident. However, no tissue carbonization was observed during the 60 s irradiations for any of the experiments.

**Figure 8:**
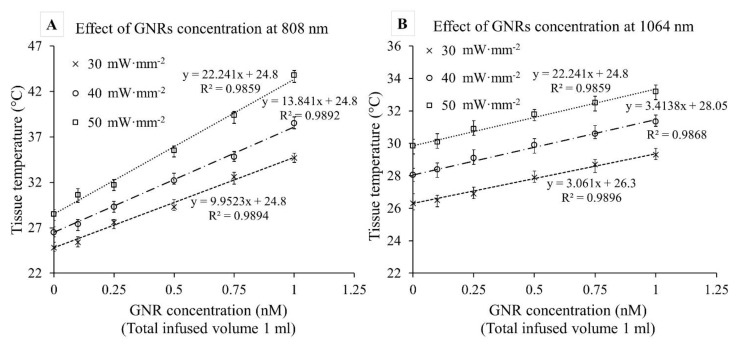
Ex vivo porcine pancreas tissue temperature vs. GNR concentration graphs for three different laser irradiations (30, 40, and 50 mW·mm^−2^) at (**A**) 808 nm and (**B**) 1064 nm wavelengths. Laser exposure time was fixed at 60 s. Error bars represent mean ± 1σ (standard deviation) for *n* = 5 trials. Statistical analysis (T-test) between tissue temperatures from different irradiations for a fixed wavelength and GNR concentration showed the significance of the data (*p* < 0.01).

Figure 8 depicts the effect of GNR concentration on ex vivo tissue photothermal heating (t = 60 s) at 808 and 1064 nm for three laser irradiances (30, 40, and 50 mW·mm^−2^). A linear trend was observed between tissue temperature rise and GNR concentration (R^2^ = 0.98). Interestingly, the gradients at 808 nm were approximately 3–5 times higher than 1064 nm (for example, at 40 mW·mm^−2^ irradiation, the slopes were 12.1 ± 0.5 °C/nM and 3.2 ± 0.3 °C/nM at 808 and 1064 nm, respectively). Slopes of the trendlines for 1064 nm were nearly identical whereas they demonstrated a slightly increasing trend for 808 nm. The steeper slope at 808 nm indicates a higher degree of temperature increase for a small increment of GNRs concentration. It is evident that the 1064 nm temperature increase overreached the 808 only when no GNRs were present, but that GNR infusion had a far more significant effect on the 808 nm irradiation. It is also worth noting that, within the laser irradiances and GNR concentrations studied, there was no visible plateauing of the heating obtained. This indicates that these experiments did not reach a saturation point, or a concentration wherein nearly 100% of the light was absorbed, and that further increases would not lead to increases in absorption.

### 3.4. Results of Computational Modelling

**Figure 9:**
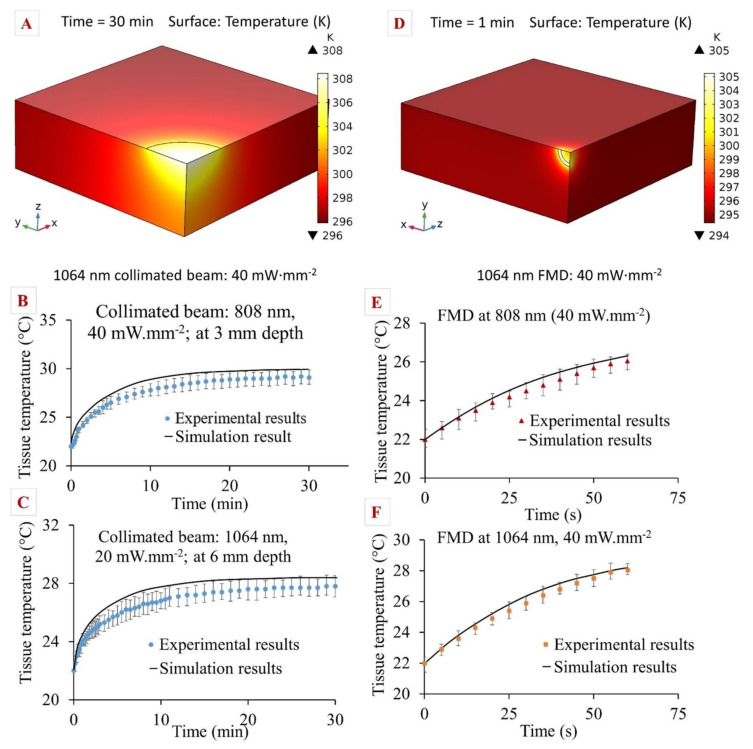
(**A**) Simulation results after 20 min of tissue photothermal heating by an 808 nm collimated beam with irradiance of 40 mW·mm^−2^; comparison between simulation and experimental results for collimated beams at (**B**) 808 nm, 40 mW·mm^−2^ irradiance, 3 mm tissue depth; (**C**) 1064 nm, 20 mW·mm^−2^ irradiance at 6 mm tissue depth; (**D**) simulation results after 1 min of tissue photothermal heating by FMD at 1064 nm, 40 mW·mm^−2^ irradiance; comparison of simulation and experimental results for FMD at (**E**) 808 nm, 40 mW·mm^−2^; (**F**) 1064 nm, 40 mW·mm^−2^ irradiances. Error bars represent mean ± 1σ (standard deviation) for *n* = 5 trials.

A computational model was developed in COMSOL Multiphysics^®^ based on the Pennes bioheat equation and the time-dependent light diffusion approximation. The model utilized tissue-specific thermal and optical parameters for the precise prediction of photon distribution and photothermal heating of pancreatic tissue. The model was modified to study the effects of two different heat sources: a collimated laser beam and light emission from the FMD tip. Tissue temperatures for 808 and 1064 nm laser wavelengths, as well as different laser irradiances, were recorded and compared against experimental results using similar conditions. Some key findings were illustrated in Figure 9 where the left-hand column (Figure 9A–C) represents the results from collimated beam exposure, and the right-hand column (Figure 9D–F) represents the results from tissue heating through the FMD tip.

In the experiments using the collimated beam, tissue temperature gradually increased until it reached a steady-state (temperature change <0.01 °C for 5 min). The temperature readings obtained from the experiments were plotted alongside the simulation results. Though the initial tissue temperatures for simulation were in close agreement with the experiments, they gradually deviated with respect to time and depth into the tissue. The difference between the theoretical and experimental data was calculated as the average of the deviation between data sets. The average deviations between both data sets were 1.2 ± 0.4 °C and 1.7 ± 0.5 °C for 808 and 1064 nm, respectively. The computational model overpredicted the temperature by 3.3 ± 0.6% and 3.7 ± 0.5% for 808 and 1064 nm, respectively. In the case of FMD, it was observed from the simulation that maximum heat was generated at the core of the fiber and the edges. The comparison between the simulation and experiment for the FMD tip followed the same procedure as the collimated beam model. The average deviations between simulation and experimental values were 4.1 ± 0.8% and 3.8 ± 0.7% for 808 and 1064 nm, respectively. 

The deviations between the simulation results and experimental values could be attributed to the optical loss due to the change in tissue physiological properties. Prior work on porcine pancreas optical properties revealed that the light transmittance and reflectance values change according to the tissue condition (fresh vs. frozen samples) [40]. Increased diffuse reflectance of frozen samples might cause less photon fluence and absorption in these experiments. These deviations, however, were relatively small as the computational model utilized tissue-specific properties for fresh/frozen samples from Akhter et al. to mimic the experimental conditions [40]. In addition, the computational model assumed all the photons from the light source were incidents on the tissue surface and propagated through the tissue. In reality, optical loss can occur during photon propagation from one medium to another due to reflection and back-scattering. Moreover, the literature review revealed a gap in C_p_ value (specific heat at constant pressure) for the porcine pancreas tissue. The specific heat for the porcine pancreas tissue at constant volume (C_v_) was reported in recent literature [52] and used in this study with the assumption that, at low temperatures (22 °C), the difference between C_p_ and C_v_ was negligible. To assess the sensitivity of the model to this parameter, additional simulations were conducted using the C_p_ value reported for the human pancreas by Agafonkina et al. [53]. These simulation results showed a higher deviation (>5%) when compared to the experimental results of the porcine pancreas tissue. This indicates that using appropriate tissue-specific properties has a considerable impact in developing accurate computational models.

Experimental results showed that the plasmonic heating of GNRs at 808 nm resulted in a rapid temperature increase due to quick light absorption and heat dissipation. Therefore, the 808 nm laser could be beneficial for PPTT, as the GNRs would absorb most of the applied laser light and cause hyperthermia to the specific tissue location while minimizing thermal damage to the healthy surrounding tissue due to the laser exposure. The effects of hyperthermia in in vivo tissue usually start at ∆T ≥ 5 °C [54]. The current study showed that, for ex vivo porcine pancreas tissue, ∆T = 5 °C was achieved within 60 s through plasmonic heating of GNRs (0.75–1 nM) with a laser irradiance of 30 mW·mm^−2^ at 808 nm. Without GNRs and with an identical laser intensity, heating of only 2 °C ∆T over 60 s was measured, indicating selective hyperthermia of the surrounding tissue can be avoided. 

Laser irradiation can be minimized by leveraging the linear relationship between tissue temperature rise and GNR concentration as shown in the current study. Higher GNR concentrations could be harmful following therapy as they tend to accumulate in the liver, spleen, and kidney and remain for a time that varies with nanoparticle size and geometry [11]. To achieve a minimum concentration, the bio-conjugation of antibodies or other targeting moieties to the surface of the GNRs to selectively localize within the target region and/or individual cells has been previously demonstrated in the literature [5,55]. Use of the FMD will also allow direct infusion within a tissue volume of interest, further reducing the amount of GNRs necessary to achieve a therapeutic effect. As the dense stroma surrounding PDAC tends to provide a barrier to local molecular transport, the 1064 nm wavelength can be used to slightly heat the targeted tissue volume and increase local diffusive, convective, and bulk transport [34,35,56,57]. However, this hypothesis requires further investigation to prove its efficacy in the pancreas tissue.

There were some limitations involved in the ex vivo tissue photothermal experiments. One of the limitations was using frozen tissue samples instead of fresh ones in some of the experiments which might affect the light absorption and photothermal heating process. Prior research revealed how thermal and optical properties change between fresh and frozen porcine pancreas tissue [40]. The current study prioritized using fresh tissue samples, but frozen samples (up to 14 days) were also included due to sourcing issues. Faster degradation of porcine pancreas tissue could affect the steady-state photothermal heating experiments. To minimize error, tissue samples were replaced after each single heating and cooling cycle. In addition, tissue samples were hydrated periodically (every 5 min) by 4–5 droplets of phosphate buffer saline (PBS) to minimize the effect of tissue dehydration during long experiments. Other limitations may be attributed to the temperature measurement techniques utilized, as they may involve error due to the resolution of the thermal camera, sensitivity, and tolerances. In addition, differences between the ex vivo conditions studied and the in vivo case should be noted. All studies were conducted at room temperature (22 °C) rather than normothermia for human tissue (~37 °C), which might affect the tissue temperature rise relative to irradiation. This is anticipated, as pancreas tissue thermal conductivity and specific heat properties are dependent on tissue temperature [52]. Moreover, as in vivo tissue has the heat sinking effects of vascular response and blood perfusion, as well as the heat generation/retention effects of metabolic activity and thermal insulation by surrounding organs, this study will need to be extended into in vivo models to properly capture the more complex response. 

Precise control of different parameters of PPTT will be critical for clinical implementation. These parameters include GNR size and concentration, laser wavelength and intensity, and the tissue thermal and optical properties. While it is necessary to start the characterization process with ex vivo experiments, the limitations for optimizing therapeutic dosage must be understood. The computational model presented will be important to further optimize the parameters and approach for this therapeutic modality. An FEM model has been presented to better understand and predict the porcine pancreas photothermal heating with 808 and 1064 nm laser wavelengths. This model accurately predicted experimental tissue temperature profiles (within a 3–5% error margin) via implementation of tissue-specific thermal and optical property measurements. The plasmonic photothermal heating model of the GNRs developed earlier by our group [24] can be coupled to this current model for predicting the PPTT in the in vivo environment. This extension of the computational model as well as the in vivo application would be the focus of future research that would facilitate the implementation of PPTT for the treatment of pancreatic cancer.

## 4. Conclusions

In this study, ex vivo porcine pancreas tissue photothermal heating was characterized with various laser conditions and concentrations of GNRs. Experiments characterized the correlations between tissue temperature and time for two different laser wavelengths. In addition, the effect of GNR concentration on tissue temperature rise was characterized through a combined infusion of laser and GNRs by FMD. A computational model was developed for tissue photothermal heating by utilizing tissue-specific thermal and optical properties. An analysis of these results identified the 808 nm laser as a potential candidate for PPTT due to the slower tissue temperature rise compared to 1064 nm, and the selective tissue heating ability through utilizing different concentrations of GNRs. These preliminary tests showed the efficacy of FMD for inducing tissue photothermal heating through the plasmonic heating of GNRs. The model developed in this study would be beneficial for future designing of PPTT for clinical application through the manipulation of different input parameters.

## 5. Patents

A technology disclosure form (TDF) was submitted entitled “An innovative intratumoral therapy for precise and local targeting of cancer tissue by utilizing a fiberoptic microneedle device for laser and liquid nanoparticle solution (i.e., gold) delivery through a diagnostic technology such as endoscopic ultras”, 2021-009. 

## Data Availability

Not applicable.

## Supplementary Materials

The following are available online at https://www.mdpi.com/article/10.3390/pharmaceutics13122133/s1, Figure S1: Relation between GNRs absorbance and concentration. The characteristic equation of the trend line was utilized to evaluate the GNRs concentration (0.1–3 nM) from the absorbance result from UV-Vis spectrometer, Figure S2: Findings of the preliminary tests to set the range of laser irradiation for ex vivo porcine pancreas tissue photothermal heating by 808 and 1064 nm laser, Figure S3: Ex vivo porcine pancreas tissue photothermal heating and free convective cooling graphs at (A) 808 nm and (B) 1064 nm wavelengths of the collimated laser beam. Four different laser irradiations were tested (20, 30, 40, 50 mW·mm^−2^) for measuring tissue temperatures at 3 and 6 mm depths. Graphs show the mean values of *n*=5 trials, Figure S4: Ex vivo porcine pancreas tissue photothermal heating by FMD utilizing both laser (808 nm; 40 mW·mm^−2^), and GNRs solution (1 nM, 1 mL) and the temperature measurement by capturing image through the thermal camera, and Table S1: Ex vivo porcine pancreas tissue optical properties for both fresh and frozen tissue sample (1 cm thick) at 808 and 1064 nm.
