# Supplementary material for: Assessment and Modeling of Plasmonic Photothermal Therapy Delivered via a Fiberoptic Microneedle Device Ex Vivo
Source: Pharmaceutics. 2021 Dec 10;13(12):2133. doi: 10.3390/pharmaceutics13122133 (PMC8703536; doi:10.3390/pharmaceutics13122133)
Supplement: Supplementary file 1 [file pharmaceutics-13-02133-s001.zip › pharmaceutics-1472997-supplementary.pdf]

pharmaceutics

Supplementary Materials: Assessment and Modeling of Plasmonic Photothermal Therapy Delivered via a Fiberoptic Microneedle Device Ex Vivo

Figure S1 was constructed by measuring the optical absorbance (at 808 nm) of a number of known concentrations of GNR solutions through the UV-Vis spectrometer. A trendline was plotted through the origin which reflects the linear relationship between optical absorbance and GNR concentrations.

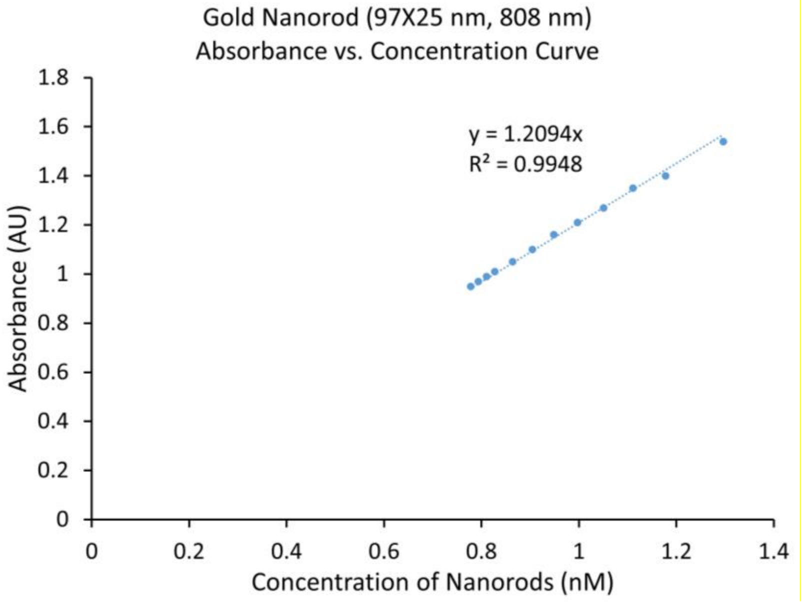

Figure S1. Relation between GNRs' absorbance and concentration. The characteristic equation of the trend line was utilized to evaluate the GNR concentration (0.1–3 nM) from the absorbance result from the UV-Vis spectrometer.

Figure S2 shows the preliminary test results to find the laser irradiation range for ex vivo porcine pancreas heating. In this test, collimated laser irradiation (both 808 and 1064 nm) was gradually increased from 10 mW·mm^{-2} with an increment of 5 mW·mm^{-2} every 60 s. Tissue temperature was monitored using a thermal camera. At the 1064 nm wavelength, when the irradiation was set at 65 mW·mm^{-2}, the tissue started to burn with visible smoke coming for the tissue surface and a rapid temperature rise to more than 100 °C. A similar condition was observed for the 808 nm wavelength, when the irradiation was set to 85 mW·mm^{-2}. After the tests, tissue samples were preserved in a formalin buffer solution for further investigation. Tissue samples were sectioned with a scalpel to investigate the penetration depth of the burning (Figure S2). It was observed that tissue burning penetrated >5 mm into the tissue thickness. FMD laser irradiation showed a similar result with carbonization at the FMD tip due to the proximity to the burned tissue (Figure S2). This carbonization at the FMD tip and on the tissue surface absorbed photon energy at a much higher rate, resulting in faster temperature rise, and propagation of burning. It can be noted that FMD can withstand a much higher laser irradiation (20 W·mm^{-2}) [32]. To avoid unwanted tissue burning, the laser irradiation range was set up to 50 mW·mm^{-2} for the current study.

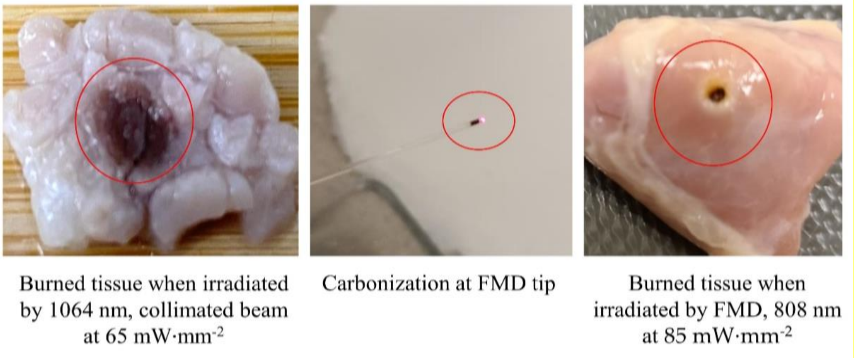

Figure S2. Findings of the preliminary tests to set the range of laser irradiation for ex vivo porcine pancreas tissue photothermal heating by 808 and 1064 nm laser.

Figure S3 shows the tissue temperature change with time when exposed to the collimated laser beam (808 and 1064 nm). The tissue sample was heated until the temperature reached a steady-state. Then, the laser was turned off to let the tissue cool down at room temperature. The sample was replaced after each single heating and cooling cycle. The same experiment was repeated with a different laser irradiation. Separate experiments were connected together to show a continuous graph of sequential heating and cooling cycles.

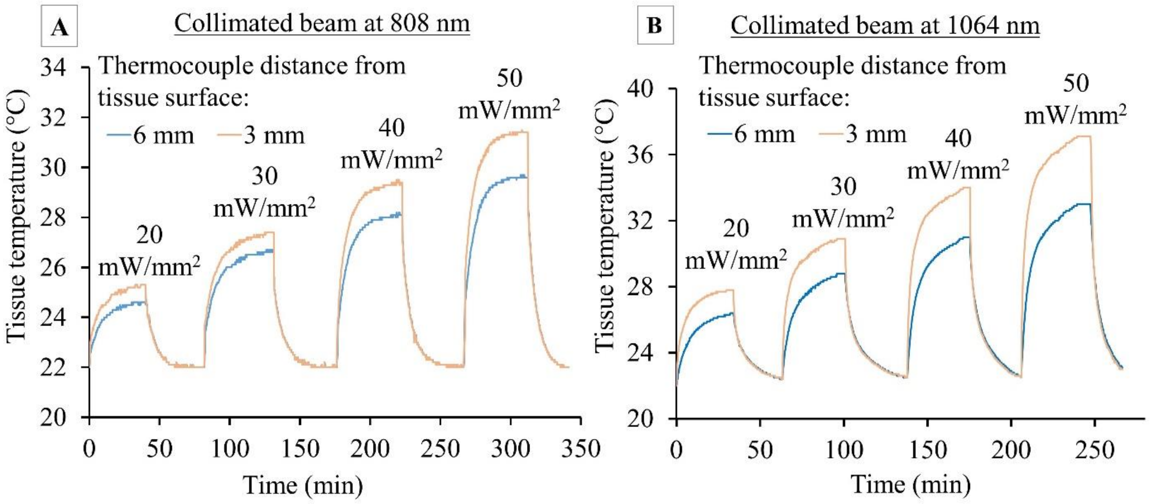

Figure S3. Ex vivo porcine pancreas tissue photothermal heating and free convective cooling graphs at (**A**) 808 nm and (**B**) 1064 nm wavelengths of the collimated laser beam. Four different laser irradiations were tested (20, 30, 40, 50 mW·mm^2) for measuring tissue temperatures at 3 and 6 mm depths. Graphs show the mean values of n = 5 trials.

Figure S4 shows the tissue temperature measurement technique through thermal camera imaging while transferring the light and GNRs' solution by FMD.

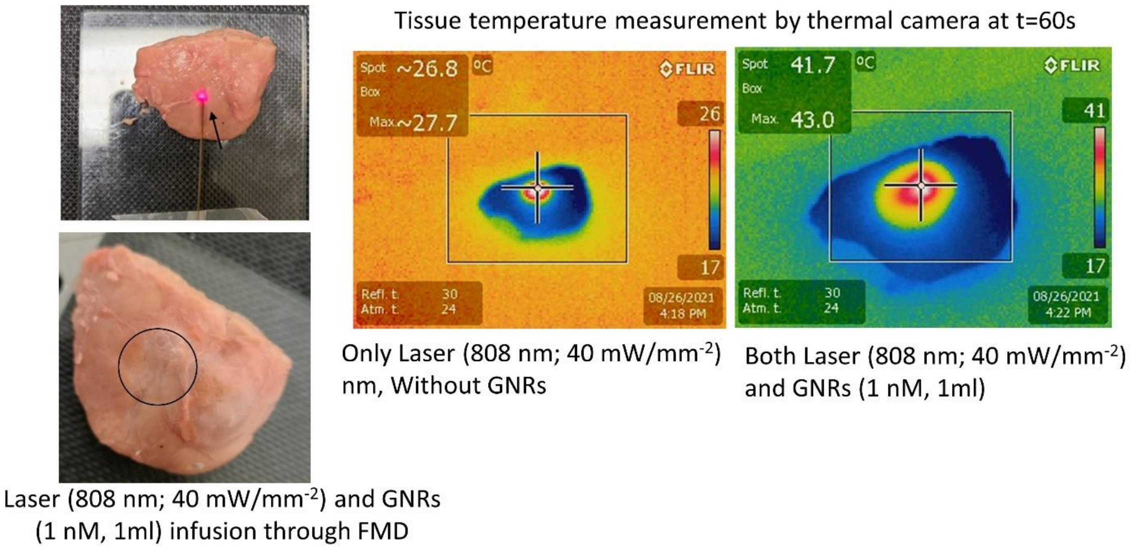

Figure S4. Ex vivo porcine pancreas tissue photothermal heating by FMD utilizing both laser (808 nm; 40 mW·mm^{-2}), and GNRs' solution (1 nM, 1ml) and the temperature measurement by capturing the image through the thermal camera.

Table S1 shows the ex vivo porcine pancreas tissue optical properties as measured by our team in an earlier study [40]. These data reflect the light absorption coefficient, reduced scattering coefficient, and light reflectance for a 1 cm thick sample.

Table S1. Ex vivo porcine pancreas tissue optical properties for both fresh and frozen tissue sample (1 cm thick) at 808 and 1064 nm.

	**Fresh Porcine Pancreas**		**Frozen Porcine Pancreas**	
Wavelength (nm)	808	1064	808	1064
Absorption coefficient (μ_a, mm^{-1})	0.013	0.057	0.015	0.11
Reduced scattering coefficient (μ_s', mm^{-1})	0.108	0.718	0.115	0.718
Light reflectance (%)	18.59	10.99	17.17	9.95
